# Influence of Institution-Based Factors on Preoperative Blood Testing Prior to Low-Risk Surgery: A Bayesian Generalized Linear Mixed Approach

**DOI:** 10.1155/2017/3624075

**Published:** 2017-12-07

**Authors:** Kazuki Ide, Hiroshi Yonekura, Yohei Kawasaki, Koji Kawakami

**Affiliations:** ^1^Department of Pharmacoepidemiology, Graduate School of Medicine and Public Health, Kyoto University, Yoshida-konoecho, Sakyo-ku, Kyoto 606-8501, Japan; ^2^Center for the Promotion of Interdisciplinary Education and Research, Kyoto University, Yoshida-honmachi, Sakyo-ku, Kyoto 606-8501, Japan; ^3^Clinical Research Center, Chiba University Hospital, 1-8-1 Inohana, Chuo-ku, Chiba 260-8677, Japan

## Abstract

To optimize delivery of health care services in clinical practice, the use of unnecessary interventions should be reduced. Although recommendations for this reduction have been accepted worldwide, recent studies have revealed that the use of such procedures continues to increase. We conducted a retrospective cohort study using a nationwide claim-based database to evaluate factors influencing preoperative blood testing prior to low-risk surgery, via a Bayesian generalized linear mixed approach. The study period was set from April 1, 2012, to March 31, 2016, and 69,252 surgeries performed at 9,922 institutions were included in the analysis. Mean patient age was 44.3 ± 11.3 years (57% female). Preoperative blood tests were performed for 59.0% of procedures. Among institutional factors, the number of beds was strongly associated with preoperative blood testing (odds ratio [95% highest posterior density interval (HPD interval)], 2.64 [2.53 to 2.75]). The difference (95% credible interval) in the rate of preoperative blood testing between institutions with <100 beds and ≥100 beds was 0.315 [0.309 to 0.322], and the Bayesian index *θ* was 1.00. This indicated that preoperative blood tests are strongly influenced by institutional factors, suggesting that specific guidelines should be developed to avoid excessive preoperative testing for low-risk surgery.

## 1. Introduction

To optimize the delivery of health care services in clinical practice, the use of unnecessary and/or non-evidence-based tests, treatments, and procedures should be reduced [1, 2]. In addition to incurring unnecessary costs, previous studies have revealed that routine preoperative tests neither decrease adverse events nor improve surgical outcomes [3–6]. In 2012, the American Board of Internal Medicine Foundation (ABIMF) launched the Choosing Wisely campaign, which encourages physicians and patients to communicate regarding the most appropriate tests and treatments for each patient [1, 7]. Since then, various countries including Japan have adopted similar campaigns [7, 8], and many specialty societies have recommended that routine preoperative testing be avoided for patients undergoing low-risk surgery [9–11]. The National Institute for Health and Care Excellence (NICE) in the UK also discourages excessive preoperative testing unless such testing yields clinical information that cannot be obtained from a patient's history and physical examination [12].

Although these campaigns and recommendations have been accepted worldwide, recent studies have revealed that the use of unnecessary tests continues to increase [13]. Previous studies have conceptualized the problem using worldwide system-level metrics to examine factors influencing the real-world situation in each country. Therefore, we conducted a retrospective cohort study using a nationwide claim-based database to evaluate factors influencing the use of preoperative blood tests prior to low-risk surgery. Specifically, we evaluated the fixed effects of the Bayesian generalized linear mixed model using Markov Chain Monte Carlo methods when examining the influence of institutional factors [14]. We also performed Bayesian conjugate analysis to compare the rate of preoperative blood testing among groups, allowing us to calculate the Bayesian index *θ*, which can be used to determine the probability that the hypothesis is true and the credible intervals (CrIs) contain the true parameter [15].

## 2. Materials and Methods

### 2.1. Study Overview

We conducted a retrospective cohort study using an insurance claim-based database covering approximately five million insured individuals in Japan since 2005. Individuals who had undergone low-risk surgery were included in the analysis. Factors influencing the use of preoperative blood tests were explored, following which institutions were divided into two groups based on the number of beds. The differences between the two groups and the probability of hypothesis truth were then evaluated.

### 2.2. Study Population

The insurance claim-based database was provided by the Japan Medical Data Center Co., Ltd. (JMDC; Tokyo, Japan), and the study period was set from April 1, 2012, to March 31, 2016. The database included the following information: sex, age, medical and pharmacy claim data (outpatient as well as inpatient data), clinical diagnostic codes (International Classification of Disease 10th revision [ICD-10]), drug prescription information codes (World Health Organization Anatomical Therapeutic Chemical classification codes [ATC codes]), and standardized procedure codes (Japan-specific standardized procedure codes [K codes]).

Low-risk surgery was defined according to K codes for ophthalmologic, superficial, breast, thyroid, minor gynecological, orthopedic [arthroscopy], and minor urological procedures, based on the findings of previous studies [9, 16]. Patients aged 20–64 years with at least 12 months of insurance data prior to undergoing low-risk surgery during the study period were included. Individuals without any required clinical data and those who had undergone low-risk procedures in an inpatient setting in conjunction with additional/emergency procedures were excluded.

### 2.3. Outcomes

The primary outcome measure of the present study was the presence or absence of preoperative blood tests prior to low-risk surgery. We used a Bayesian generalized linear mixed approach to estimate the coefficients of each variable (patient variables and institutional factors) for preoperative blood tests. Preoperative tests were defined as those ordered within 60 days of the index procedure [17] and included the following: complete blood count (CBC), basic metabolic panel, coagulation tests, and liver function tests (LFTs). In Japan, health insurance coverage of these preoperative tests does not differ based on the patient's health insurance status. Institutional differences in preoperative testing rates were regarded as the secondary outcome measure.

### 2.4. Statistical Analysis

Continuous variables are summarized as mean and standard deviations (SD), while categorical variables are summarized as frequencies and proportions (%).

In the present study, we evaluated differences in preoperative testing among institutions using a Bayesian generalized linear mixed model [14, 18]. Institutions were regarded as random effects, and the model contained two levels of hierarchical structure.

In this model, *Y*_*ij*_ indicates whether the *j*th individual in the *i*th institution had undergone preoperative blood testing. Thus, *Y*_*ij*_ represents the Bernoulli distribution of parameter *π*_*i*_. The rate *π*_*i*_ represents the rate of preoperative blood testing at each institution. We assumed that the random effect *c* had a mean of 0 and a normal distribution for variance *σ*_0_^2^. The hyperparameter *σ*_0_^2^ was assumed to follow a gamma distribution, which does not take negative values, as it represents the variance. The shape and scale parameters of the gamma distribution were each set to 0.001, assuming a fixed effect with a mean of 0 and a normally distributed variance of 10,000. Using these parameters, the model can be expressed as follows:

(1) log⁡πi1−πi=β0+β1x1ij+β2x2ij+⋯+βkxkij+⋯+βpxpij+c,βkij~N0,1000,c~N0,σ02,σ02~Gamma0.001,0.001,

where *β*_*k*_ represents the fixed-effect parameter and *x*_*kij*_ represents the *j*th individual in the *i*th institution. All patient variables (age [reference: <25 years], sex [reference: male], Charlson comorbidity index [CCI; reference: 0-1], antiplatelet use [reference: nonuse], anticoagulant use [reference: nonuse], angiotensin-converting enzyme inhibitor/angiotensin-receptor blocker use [reference: nonuse], diuretic use [reference: nonuse], chemotherapy [reference: nonchemotherapy], type of anesthesia [reference: local anesthesia], ophthalmologic procedure [reference: nonophthalmologic procedure], and surgical setting [reference: inpatient setting]) and institutional factors (number of beds [reference: <100], hospital status [reference: nonteaching hospital], and number of operations [reference: Q1 {lowest}]) were included as predictors in the Bayesian generalized linear mixed model. We used the aforementioned expression to calculate the posterior mean and the standard error of each parameter as well as the 95% highest posterior density (95% HPD) interval. We calculated the posterior distribution using Markov Chain Monte Carlo methods by applying the Metropolis-Hastings method to the calculation algorithm. A total of 20,000 samples were calculated, and the first 500 samples were discarded as burn-in. We used graphical plots to interpret the convergence of the MCMC results [19]. The posterior mean of each parameter was used as the model coefficient, and these values were expressed as odds ratios (exp⁡[*β*_*k*_] = odds ratio).

We calculated the number of preoperative blood tests and compared the rate of testing between institutions with <100 beds and ≥100 beds. We calculated the 95% credible interval (CrI) of the difference in the rate of preoperative blood testing using an exact method. We then compared rates of preoperative blood testing between the two institution groups using the Bayesian index proposed by Kawasaki and Miyaoka [15]. The threshold of 100 beds has previously been used as an institutional criterion for categorizing general hospitals in Japan, and a recent survey in Japan has also demonstrated the medical relevance of this threshold [20]. Therefore, we chose to use a threshold of 100 beds.

In the present study, we set *z*_1_ as the number of preoperative blood tests at institutions with <100 beds and *z*_2_ as that for institutions with ≥100 beds. We regarded *Z*_1_ and *Z*_2_ as binomial random variables for *n*_1_ preoperative blood tests and *n*_2_ preoperative blood tests and parameters *p*_1_ and *p*_2_, respectively. The conjugate prior density for *p*_1_ is the beta distribution with parameters *α*_*i*_ and *β*_*i*_, with parameters *α*_*i*_ > 0, *β*_*i*_ > 0, and *i* = 1, 2. The prior distribution was regarded as noninformative and defined as beta (1, 1). The posterior pdf for *p*_1_ is the proposed beta distribution with parameters *a*_*i*_ = *α*_*i*_ + *z*_*i*_ and *b*_*i*_ = *n*_*i*_ − *α*_*i*_ + *β*_*i*_. Using the posterior pdf, we calculated the exact Bayesian index *θ*. The accurate expression of probability for Bayesian index *θ* is proposed as follows:

(2) Pp1<p2 ∣ z1,z2=Ba1+a2,b1a2Ba1,b1Ba2,b2·F23a2,1−b2,a1+a21+a2,a1+a2+b1;1,

where F23k1,k2,k3l1,l2;1=∑t=0∞(k1)t(k2)t(k3)t/(l1)t(l2)t1/t!,  *k*_1_ + *k*_2_ + *k*_3_ < *l*_1_ + *l*_2_, represent hypergeometric series and (*k*)_*i*_ is the Pochhammer symbol.

All statistical analyses were performed using SAS version 9.4 for Windows (SAS Institute Inc., Cary, NC, USA). When performing the analysis using the Bayesian generalized linear mixed model, we used the MCMC procedure of SAS. A general SAS code for this analysis is included in Appendix 2.

### 2.5. Ethical Considerations

The present study was approved by the Ethics Committee of Kyoto University Graduate School and Faculty of Medicine (number R0800, September 8, 2016). This study was conducted in accordance with the Declaration of Helsinki and the Ethical Guidelines for Medical and Health Research Involving Human Subjects in Japan [21, 22]. Additional informed consent was not required based on these guidelines. The Strengthening the Reporting of Observational Studies in Epidemiology (STROBE) criteria were applied in the reporting of the present study [23].

## 3. Results

### 3.1. Clinical Characteristics of Participants

**Figure 1:**
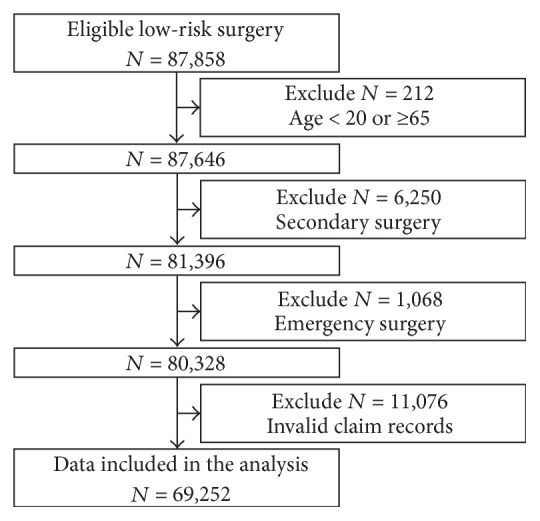
Study flow diagram.

The flow diagram for the present study is shown in Figure 1, and patient characteristics are presented in Table 1.

During the study period, 3,543,575 individuals from 65,371 institutions were included in the JMDC database. Among them, 70,244 individuals had undergone a total of 87,858 low-risk surgical procedures. Following the exclusion of 18,606 cases that met exclusion criteria, 69,252 surgeries performed at 9,922 institutions were included in the final analysis. Mean age (SD) in this cohort was 44.3 years (11.3 years), and 57.0% of patients were female. Local anesthesia was most frequently performed. Inpatient procedures accounted for 31.0% of surgical cases, and 40.6% of institutions had ≥100 beds.

### 3.2. Rate of Preoperative Blood Testing and Bayesian Generalized Linear Mixed Approach

Preoperative blood tests were performed for 59.0% of procedures. The prevalence of each preoperative blood test was as follows: CBC, 57.8%; basic metabolic panel, 49.6%; LFTs, 48.0%; coagulation test, 35.6%. The odds ratio of each variable (patient variables and institutional factors) with 95% HPD intervals for preoperative blood testing is presented in Table 2. Among patient variables, general anesthesia (5.42 [4.85 to 6.03]), anticoagulant use (3.57 [2.22 to 5.61]), and regional anesthesia (3.14 [2.89 to 3.44]) were relatively associated with preoperative blood testing. Among institutional factors, the number of beds within an institution was strongly associated with preoperative blood testing (odds ratio [95% HPD interval], 2.64 [2.53 to 2.75]). The graphical plots used to interpret the convergence of the MCMC results are included in Appendix 1.

### 3.3. Differences in Preoperative Blood Testing and Bayesian Index

The posterior beta distribution parameters were *a*_1_ = 21829 + 1 = 21830 and *b*_1_ = 28095 − 21829 + 1 = 6267 for institutions with ≥100 beds and *a*_2_ = 18993 + 1 = 18994 and *b*_2_ = 41157 − 18993 + 1 = 22165 for institutions with <100 beds. The difference [95% CrI] in the rate of preoperative blood testing between institutions with <100 beds and ≥100 beds was 0.315 [0.309 to 0.322], and the Bayesian index *θ* was 1.00 (Table 3). These findings indicate a point estimate of a 31.5% difference in preoperative blood testing, with a true difference between 30.9% and 32.2%. Based on the value of the Bayesian index *θ*, this difference can be observed as 100%.

## 4. Discussion

The present study aimed to evaluate factors influencing preoperative blood testing prior to low-risk surgery among individuals in a nationwide claim-based database using Bayesian approaches. Our results indicated that the rate of preoperative blood testing is strongly influenced by institutional factors, such as institution size. Furthermore, our results suggested that patient factors were also associated with preoperative blood tests. However, the influence of institutional factors remained after adjusting for these variables, indicating that modification of practices at the institutional level is necessary to reduce unnecessary preoperative blood testing.

In this study, we utilized a nationwide claim-based database covering 4.7 million insured individuals treated at more than 9,000 institutions in Japan [24], suggesting that our results may be highly generalizable. As we adopted a Bayesian approach with the institutions set as random effects, our results regarding the dispersion of this parameter can also be extrapolated to real-world settings. Our Bayesian generalized linear mixed model approach indicated that the adjusted odds ratio for institutions with ≥100 beds was >2.5 and that the number of beds was the most influential of the institutional factors investigated. The convergence of the MCMC results is slightly challenging because of strong autocorrelation. Thinning, or other such techniques (e.g., extension of the chain) with at least two or three parallel chains per parameter, can improve the accuracy of the analysis [25]. However, these results were consistent with those of an additional univariate model analysis (OR [95% HPD interval], 4.06 [3.93 to 4.21]), highlighting the robustness of our finding (univariate model analysis for other predictors are included in Appendix 3). Furthermore, a Bayesian association analysis among predictors which was not planned for our current study may be able to improve the impact of our analysis [26, 27].

The 95% CrI for differences between institutions with <100 beds and ≥100 beds was very narrow (approximately 1%). The use of Bayesian approaches for the calculation of the CrI is advantageous in that the true parameter is contained within the interval [28, 29]. Thus, our findings strongly suggest that institutional factors influence that rate of preoperative blood testing, consistent with the findings of previous Canadian studies [9, 17]. Institutions with a large number of beds frequently performed preoperative blood tests, which may have been due to the following reasons: (1) institutions with a large number of beds have enough resources to perform routine laboratory tests regardless of patient status and (2) such testing may be routine at larger institutions. Our findings suggest that clear guidelines for preoperative blood testing are required to avoid unnecessary laboratory tests.

The present study possesses several limitations of note. The main limitation of this study was the use of a database with limited information, as the database did not include information regarding symptoms or physical examination results. In addition, the number of operations could be only analyzed as quantile-categories. Therefore, we were unable to strictly evaluate the suitability of preoperative blood tests for each patient in the present study. In addition, the database contained information from a limited population of participants. Because the database contained only insurance claim-based data accumulated from medium-to-large scale companies in Japan, it only included data for employees under the age of 75 and their families. Therefore, we were unable to examine the influence of patient age on preoperative blood testing. Indeed, previous studies have reported that advanced age is a risk factor for perioperative events and complications, even in low-risk surgeries [30]. Additional investigations involving older adults are thus required to more fully elucidate the influence of patient-based and/or institutional-based factors on the rate of preoperative blood testing.

## 5. Conclusion

In conclusion, our findings indicate that preoperative blood testing prior to low-risk surgery is influenced by institutional factors, such as institution size, suggesting that Bayesian approaches can be used to develop guidelines aimed at reducing excessive preoperative testing. Future studies should investigate the influence of additional patient characteristics (e.g., age) in a more varied population in order to establish the most appropriate guidelines.

## Figures and Tables

**Table 1:** Patient characteristics.

Characteristics	
*N*	69,252
Age, yr	
<25	3,070 (4.4)
25–34	13,172 (19.0)
35–44	19,816 (28.6)
45–54	18,281 (26.4)
55–64	14,913 (21.5)
Sex, female	39,489 (57.0)
CCI score	
0-1	57,106 (82.5)
2	6,742 (9.7)
≥3	5,404 (7.8)
Medication	
Antiplatelet	1,759 (2.5)
Anticoagulant	330 (0.5)
ACEI/ARB	5,399 (7.8)
Diuretics	1,075 (1.6)
Chemotherapy	792 (1.1)
Type of anesthesia	
General anesthesia	8,824 (12.7)
Regional anesthesia	7,291 (10.5)
Sedation	7,802 (11.3)
Local anesthesia	38,874 (56.1)
Unknown	6,461 (9.3)
Surgical setting	
Inpatient	21,496 (31.0)
Outpatient	47,756 (69.0)
Teaching hospital	5,372 (7.8)
Number of beds	
<100	41,157 (59.4)
≥100	28,095 (40.6)

ACEI, angiotensin-converting enzyme inhibitor; ARB, angiotensin-receptor blocker; CCI, Charlson comorbidity index.

**Table 2:** Odds ratio for patient and institutional factors.

Variables	OR [95% HPD interval]
*Patient factors*	
Age	1.09 [1.07 to 1.11]
Sex (female)	1.03 [0.99 to 1.07]
CCI	1.84 [1.77 to 1.92]
Antiplatelet	1.40 [1.23 to 1.63]
Anticoagulant	3.57 [2.22 to 5.61]
ACEI/ARB	1.53 [1.42 to 1.66]
Diuretics	1.40 [1.15 to 1.69]
Chemotherapy	1.55 [1.16 to 2.09]
Type of anesthesia	
General anesthesia	5.42 [4.85 to 6.03]
Regional anesthesia	3.14 [2.89 to 3.44]
Sedation	2.19 [2.05 to 2.34]
Unknown	0.63 [0.58 to 0.66]
Ophthalmologic procedure	1.58 [1.47 to 1.69]
Outpatient	0.37 [0.35 to 0.39]

*Institutional factors*	
Hospital with ≥100 beds	2.64 [2.53 to 2.75]
Teaching hospital	0.71 [0.66 to 0.77]
Number of operations	1.15 [1.13 to 1.17]

CEI, angiotensin-converting enzyme inhibitor; ARB, angiotensin-receptor blocker; CCI, Charlson comorbidity index; HPD, highest posterior density; OR; odds ratio. *Notes*. The posterior mean of each parameter was used as the model coefficient, and these values were expressed as odds ratios (exp⁡(*β*_*k*_) = odds ratio).

**Table 3:** Differences in the rate of preoperative blood testing based on institution size.

	Preoperative blood test		Total
	−	+	
Number of beds			
<100	22164 (53.9)	18993 (46.1)	41157
≥100	6266 (22.3)	21829 (77.7)	28095

Difference (95% CrI)			*θ*

0.315 [0.309 to 0.322]			1.00

CrI, credible interval; *θ*, Bayesian index.

## Supplementary Materials

Supplementary Materials Appendix  1. MCMC trace and MCMC autocorrelation function plots. Appendix  2. SAS code for analysis. Appendix  3. Univariate analysis for patient and institutional factors.
