# Supplementary material for: Influence of Institution-Based Factors on Preoperative Blood Testing Prior to Low-Risk Surgery: A Bayesian Generalized Linear Mixed Approach
Source: Comput Math Methods Med. 2017 Dec 7;2017:3624075. doi: 10.1155/2017/3624075 (PMC5738628; doi:10.1155/2017/3624075)
Supplement: Supplementary Materials — Appendix 1. MCMC trace and MCMC autocorrelation function plots. Appendix 2. SAS code for analysis. Appendix 3. Univariate analysis for patient and institutional factors. [file 3624075.f1.pdf]

Appendix 1. MCMC trace and MCMC autocorrelation function plots

Age

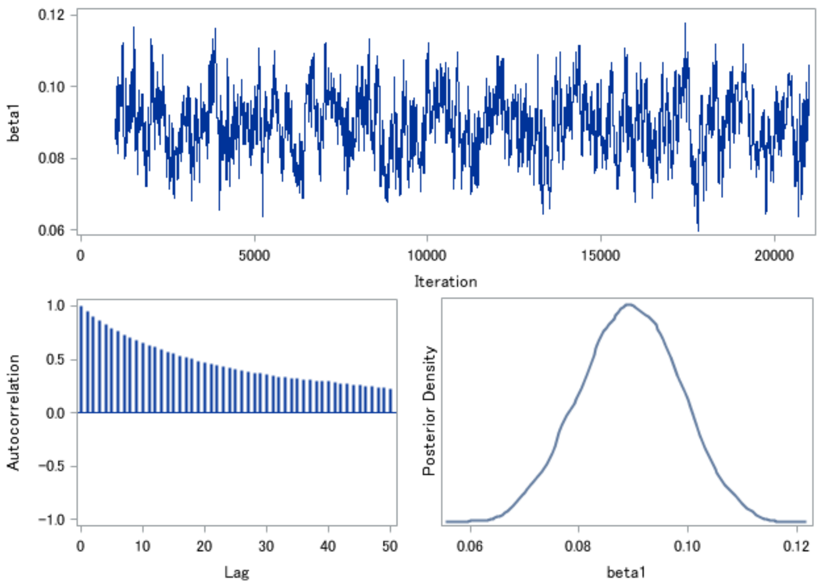

Sex

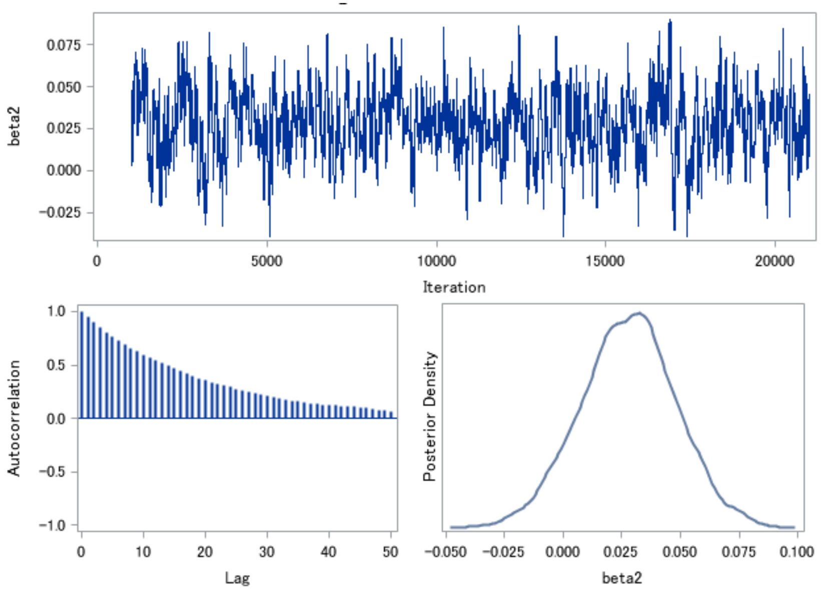

Number of beds

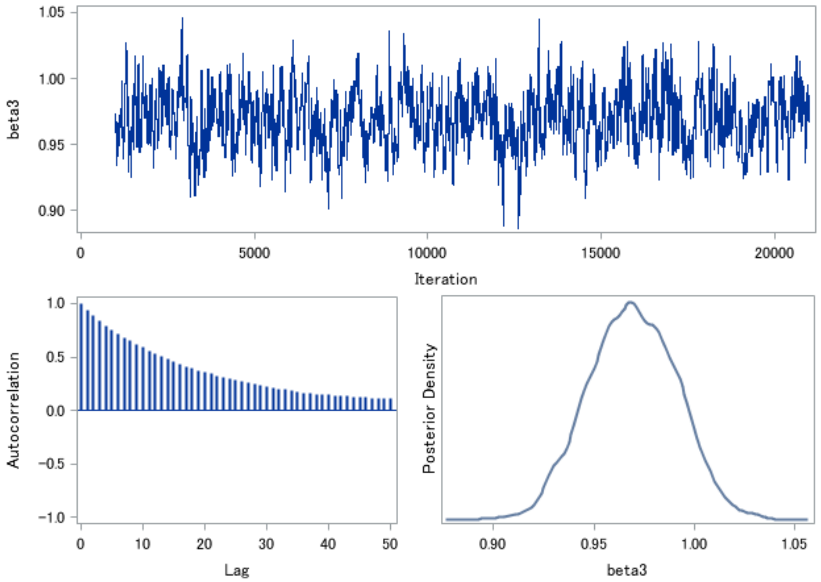

Hospital status

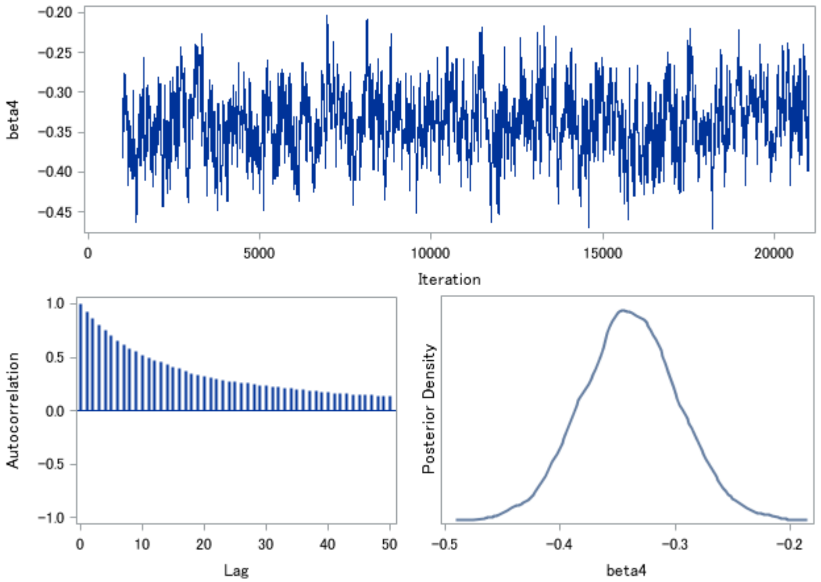

Appendix 1. *Count*.

Surgical setting

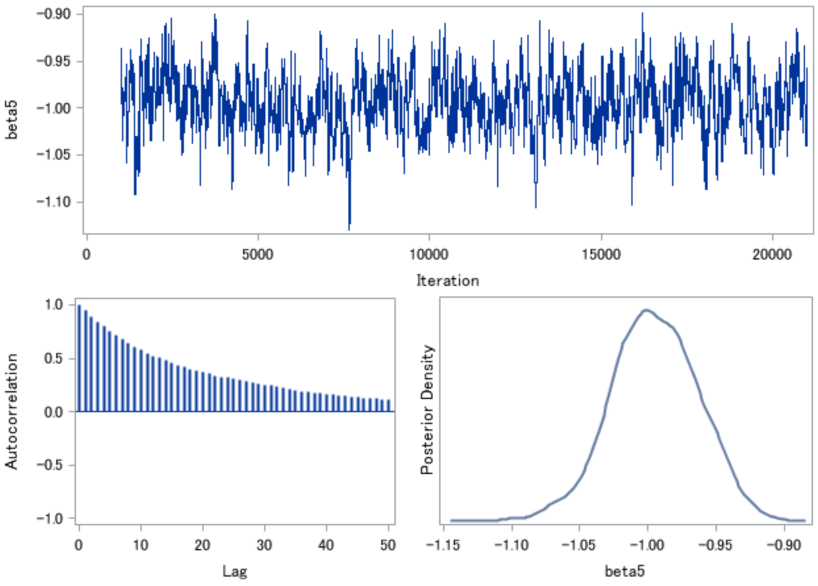

Charlson comorbidity index

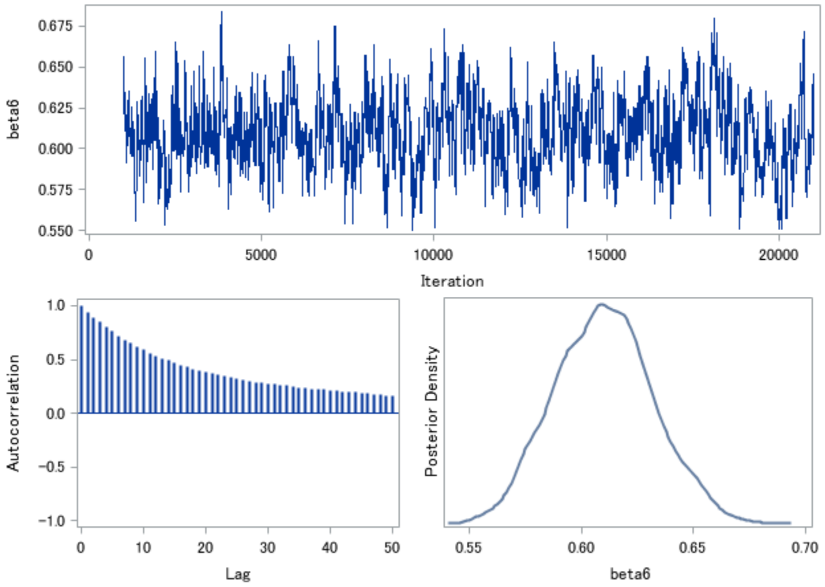

Anti-platelet use

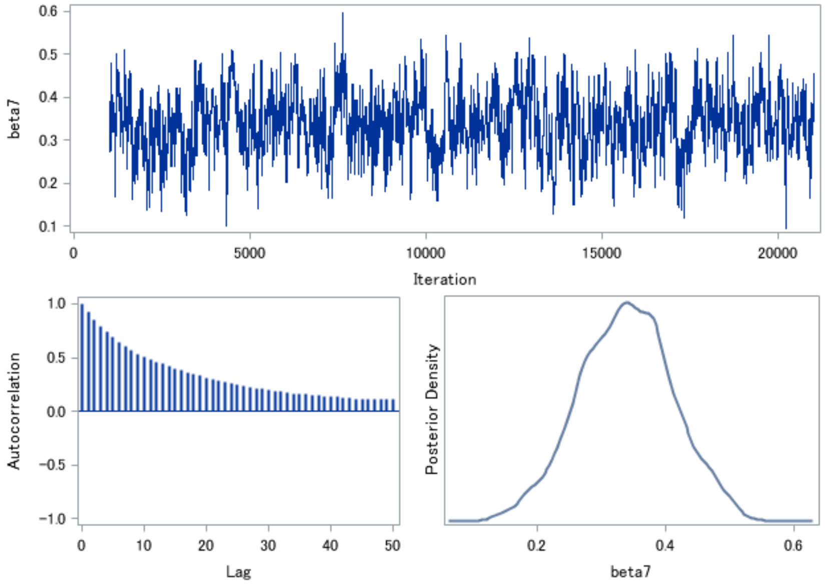

Anticoagulant use

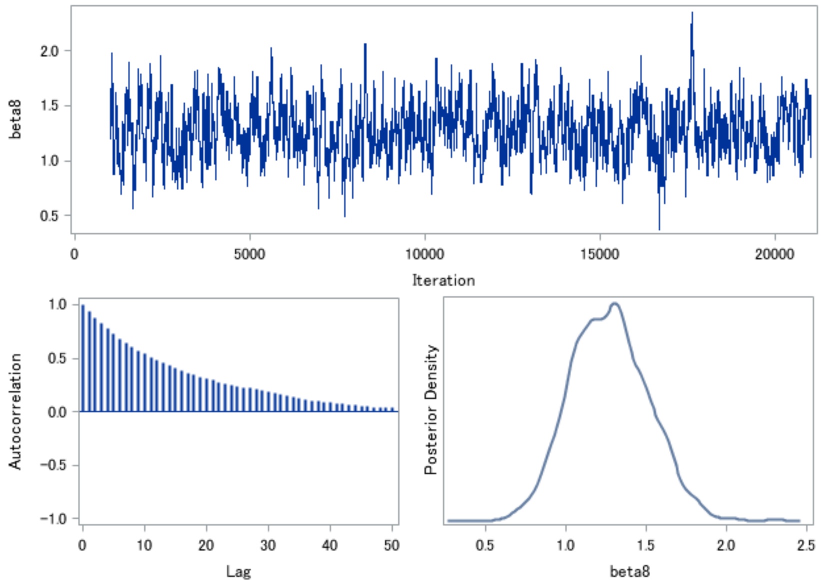

Appendix 1. *Count*.

ACEI/ARB

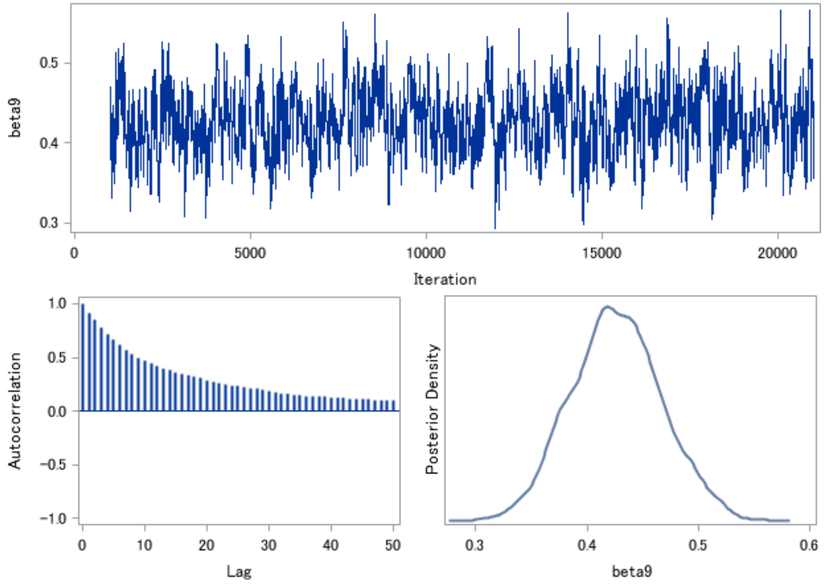

Diuretics use

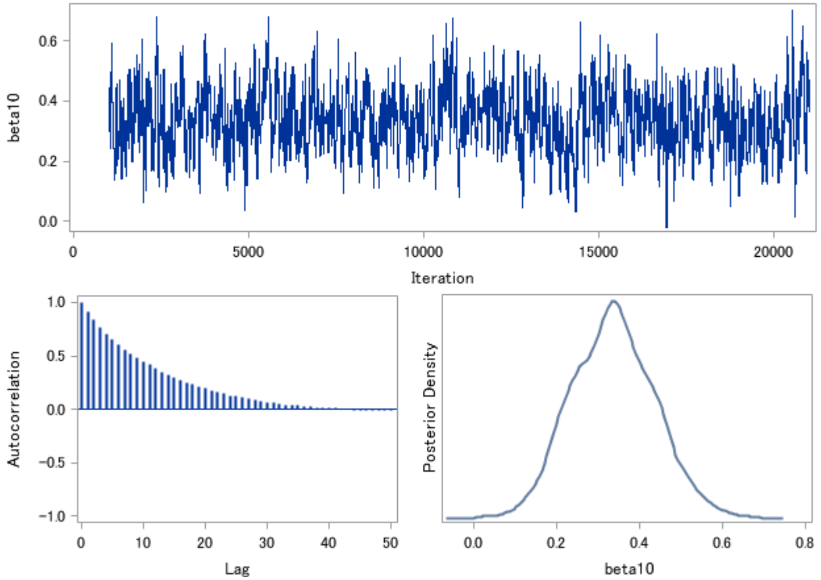

Chemotherapy

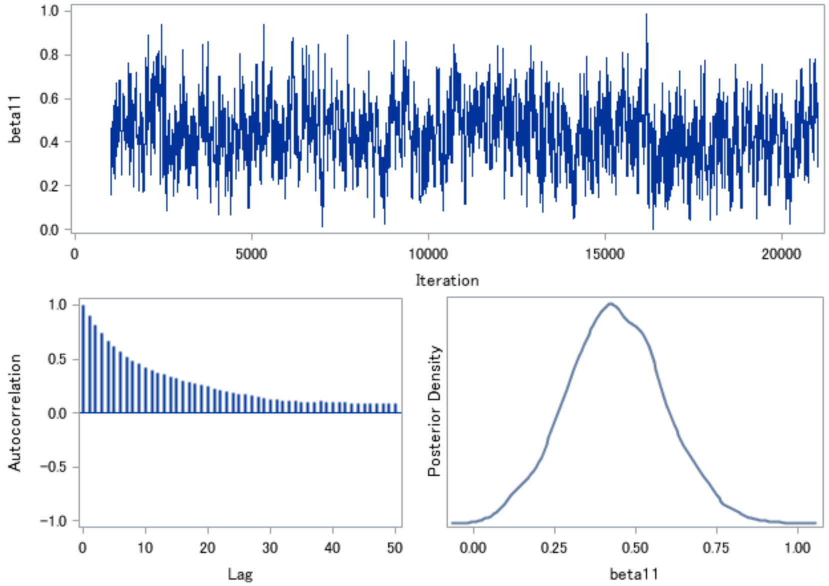

General anesthesia

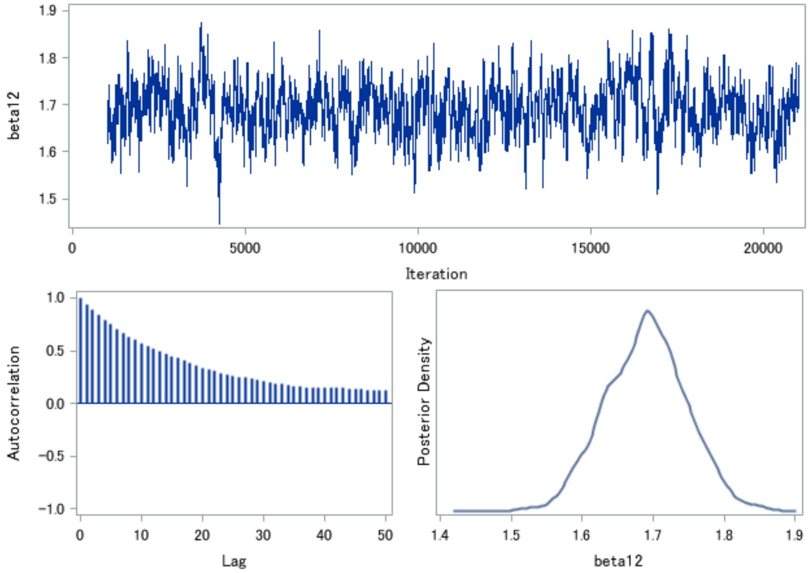

Appendix 1. *Count*.

Regional anesthesia

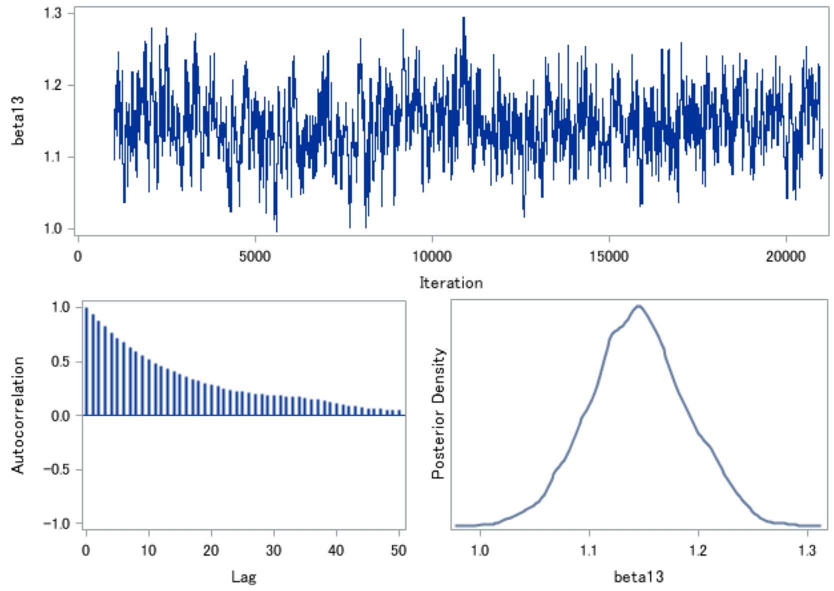

Sedation

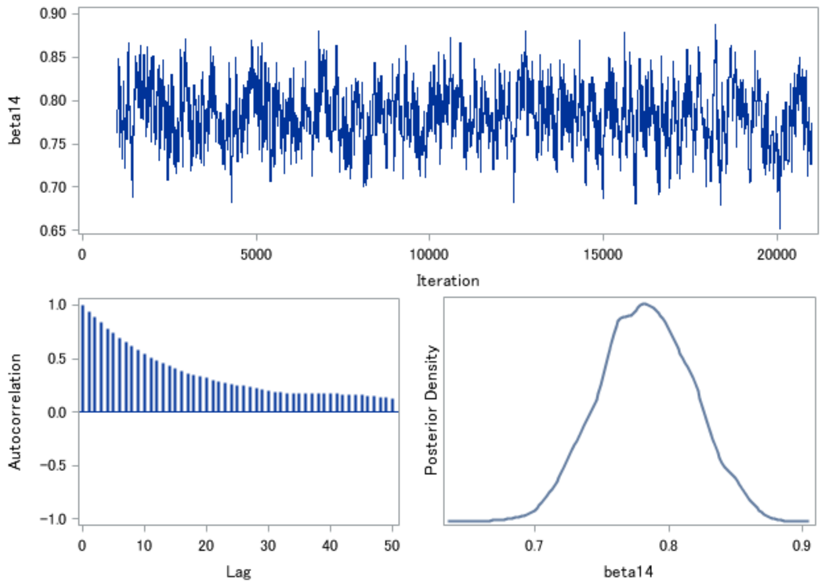

No information (anesthesia)

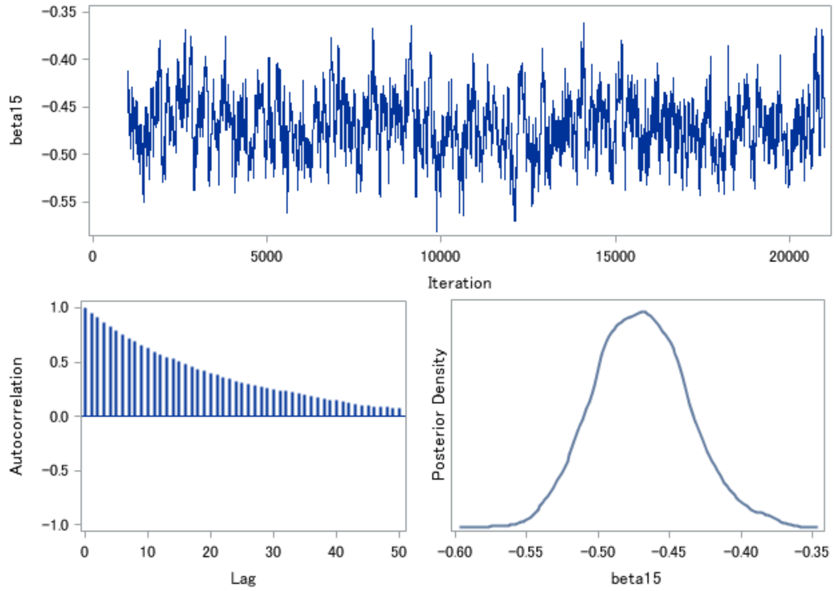

Ophthalmologic procedure

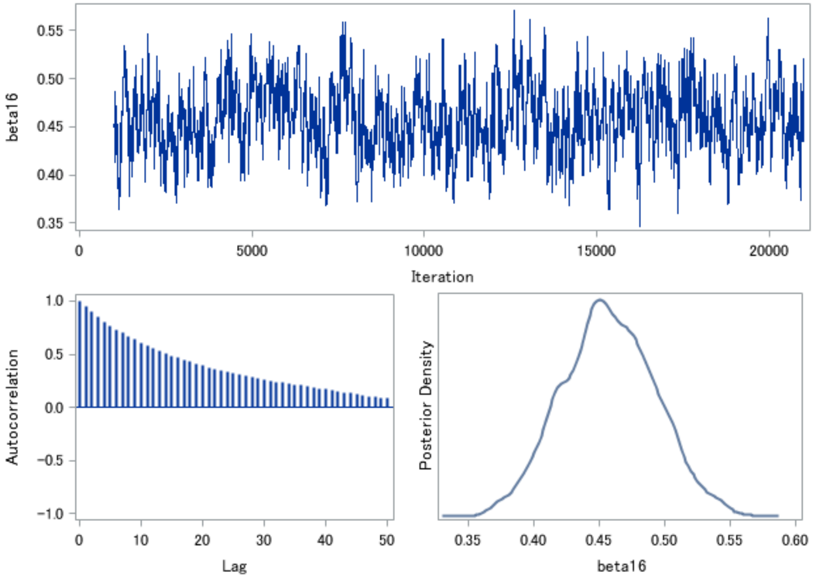

Appendix 1. *Count*.

Number of operation (Quantiles)

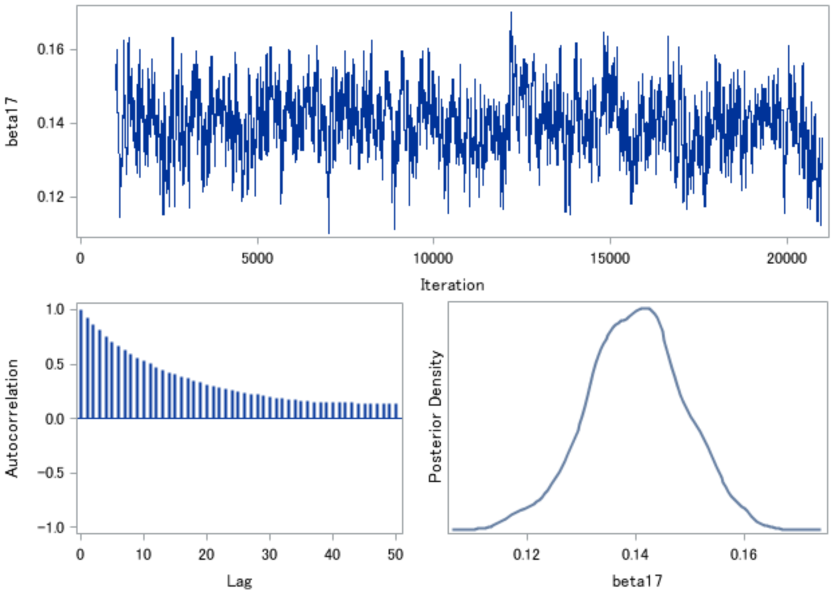

Appendix 2. SAS code for analysis

**** Bayesian generalized linear mixed model (MCMC, general SAS code)*;**

```
proc mcmc data = work.DATA01 ntu = 1000 nmc = 20000 nthin = 2 propcov = quanew
    outpost = KENSA seed = 246810 dic;
    ods select PostSumInt TADpanel;
    parms (alpha beta1) 0; *beta =fixed-effect parameter;
    parms c 1; *c = random effect parameter;
    prior alpha beta1 ~ normal (0, var = 10000);
    p = logistic (alpha + beta1*A); *A = predictor(s), it is depending on the number of predictors;
    prior c ~ gamma (0.001, scale = 0.001);
    random u ~ normal (0, var = c) subject=ID_SITE; *ID_SITE=Site ID;
    model cccl ~ binomial (1, p); *cccl= preoperative blood tests prior to low-risk surgery;
run;
```

Appendix 3. Univariate analysis for patient and institutional factors

Variables	OR [95% HPD interval]
Patient factors	
Age	1.21 [1.20 to 1.23]
Sex (female)	1.03 [1.00 to 1.07]
CCI	2.73 [2.64 to 2.83]
Anti-platelet	2.83 [2.51 to 3.17]
Anticoagulant	8.69 [5.63 to 12.9]
ACEI/ARB	2.32 [2.17 to 2.47]
Diuretics	3.98 [3.40 to 2.47]
Chemotherapy	7.58 [5.90 to 9.75]
Type of anesthesia	
General anesthesia	15.3 [13.9 to 16.9]
Regional anesthesia	5.54 [5.15 to 5.91]
Sedation	1.49 [1.42 to 1.56]
Unknown	0.44 [0.42 to 0.46]
Ophthalmologic procedure	2.01 [1.92 to 2.11]
Outpatient	0.12 [0.11 to 0.13]
Institutional factors	
Hospital with ≥100 beds	4.06 [3.93 to 4.21]
Teaching hospital	2.91[2.72 to 3.12]
Number of operation	1.32 [1.30 to 1.33]

CEI, angiotensin-converting enzyme inhibitor; ARB, angiotensin-receptor blocker; CCI, Charlson comorbidity index; HPD, highest posterior density; OR; odds ratio. Notes: The posterior mean of each parameter was used as the model coefficient, and these values were expressed as odds ratios (exp (β_k) = odds ratio).
